# Investigating the relationship between health literacy and preconceptual care components during the first 14 weeks of pregnancy: a cross-sectional study

**DOI:** 10.1186/s12875-024-02467-5

**Published:** 2024-06-11

**Authors:** Fatemeh Sajjadian, Leila Amiri-Farahani, Shima Haghani, Sally Pezaro

**Affiliations:** 1 Department of Reproductive Health and Midwifery, Nursing and Midwifery Care Research Center, School of Nursing and Midwifery, Iran University of Medical Sciences, Tehran, Iran; 2 Department of Biostatistics, Nursing and Midwifery Care Research Center, Iran University of Medical Sciences, Tehran, Iran; 3 The Research Centre for Healthcare and Communities, Coventry University, Coventry, UK; 4 The University of Notre Dame, Notre Dame, Australia

**Keywords:** Health literacy, Preconceptual care, Pregnancy, Folic acid consumption, Preventative healthcare

## Abstract

**Background and Aim:**

Preconceptual care aiming to improve health is influenced by various factors including health literacy. Considering the importance and necessity of high quality preconceptual care, this study aimed to determine the relationship between health literacy and receiving components of preconceptual care prior to pregnancy.

**Methods:**

This cross-sectional study included 693 participants with pregnancies of less than 14 weeks gestation referred to health centers and gynecologists in Shiraz city, Iran. Multi-stage sampling was done from May 2021 to February 2022 in 18 comprehensive urban health centers and 20 gynecology offices via proportional allocation method. The data collection tool comprised a questionnaire consisting of 3 parts: (1) individual and fertility characteristics, (2) information related to the components of preconceptual care and (3) health literacy for Iranian adults. This was completed by individual participants via the self-reporting method.

**Results:**

The majority of participants were between 30 and 34 years old. They also identified as women with a university education and were predominantly unemployed. The mean health literacy of participants was 76.81%. Health literacy obtained the highest mean score in the dimension of ‘understanding’ and the lowest mean score in the dimension of ‘access’. The frequency of preconceptual counseling, folic acid supplement consumption, exercise, blood testing, dental visits, genetic counseling, Pap smear testing and rubella, diphtheria, and hepatitis vaccinations prior to pregnancy was 66.8%, 53.8%, 45.6%, 71.86%, 44.44%, 12%, 53.4%, 10.83%, respectively. Many (> 64%) received preconceptual care at specialist gynecology offices. Results demonstrated that health literacy had a statistically significant relationship with preconceptual care, folic acid consumption, exercise and dental care, (*p* < 0.001), along with blood testing and Pap smear testing (*p* < 0.05).

**Conclusion:**

Overall, our results demonstrate that despite health literacy being optimal, uptakes of some components of preconceptual care are low. As such, it will be important to further raise awareness of the importance of preconceptual care for people prior to pregnancy as a priority in health promotion and education.

**Supplementary Information:**

The online version contains supplementary material available at 10.1186/s12875-024-02467-5.

## Introduction

Preconceptual care (PCC) is carried out with those of reproductive age, prior to becoming pregnant or in the interval between pregnancies. PCC is considered necessary to promote health and wellbeing during pregnancy and childbirth [1, 2]. It leads to improved diet, increased folic acid supplementation, reduced smoking, weight loss and improved management of diabetes, all of which enhance perinatal outcomes [3–6]. According to the World Health Organization, PCC also reduces the occurrence of unwanted pregnancy by 50% [7], thus further reducing the risk of adverse perinatal outcomes [8]. PCC is particularly important in a context where perinatal mortality occurs most frequently in those who report not having had it [9, 10]. Nevertheless, the frequency of receiving PCC varies globally. For example, in the United States of America (USA), only one-third receive PCC [11]. Elsewhere, 40% in China [12], 18.2% in Ethiopia [13], 15.8% in France [14], 15.9% in Brazil [15], 27.2% in southern Sri Lanka [16] and 2.5% in Nigeria [17] receive PCC. In Iran, the prevalence of PCC differs from city to city, as 34.2% receive PCC in Mashhad [18] and 47.7% receive PCC in Isfahan [19]. Considering the above, there is considerable scope to improve both the provision and uptake of PCC on an international scale, particularly as considering the above, its relevance to primary care is clear.

Various factors are related to receiving PCC, including the availability of public and private health centers [10, 14], the cost of some services [20], treatment of healthcare workers [20], family support [13], access to information sources [21], age, education level, socio-economic status [11], chronic disease history [13], number of births [14] and health literacy [22, 23]. Health literacy refers to a person’s ability to evaluate, understand and use health-related information that promotes and maintains health [23, 24]. Thus, health literacy is closely related to preventive and health-promoting behaviors [25, 26]. Nevertheless, levels of health literacy are reportedly low [27]. In eight European countries 59% of people had insufficient health literacy [28]. In Iran, 44% of people reportedly have limited health literacy [29], with levels of health literacy being particularly low in Shiraz [30]. This is significant, as low levels of health literacy can result in people being less likely to receive PCC [31], including preconceptual counseling, folic acid supplementation, and vaccination [32]. Conversely, those who have higher levels of health literacy are more likely to receive preventive measures related to childbearing [33]. Consequently, it will be important to explore these relationships further and identify primary care strategies for increased health literacy and uptake of PCC globally.

Results regarding the relationship between health literacy and the receiving of preventive health services are contradictory [34, 35]. Moreover, no studies to date have explored the relationship between health literacy and PCC specifically, particularly in geographical areas where significantly low levels of healthcare literacy are reported, such as in Shiraz, Iran [30]. For this reason, this study aimed to determine the relationship between health literacy and the receiving of PCC including participants referring to urban health centers and the offices of gynecologists in Shiraz, Iran.

## Methods

### Study design and setting

This cross-sectional study was conducted with participants referring to health centers and gynecology offices in Shiraz, Iran. The selection of urban health centers as study settings was done using a multi-stage method. In the first stage, health centers were divided into 5 clusters based on the socio-economic level of potential participants in the Shiraz Municipality. The first cluster was classified as ‘high level’ (6 centers), the second cluster consisted of ‘upper middle level’ (5 centers), the third cluster consisted of ‘normal level’ (11 centers), the fourth cluster was classified as ‘average downward’ (7 centers) and the fifth cluster was classified as ‘weak’ (6 centers), from which 50% of the centers were selected via a simple random method. From all private gynecology offices in Shiraz (*n* = 165), 20 were selected via a simple random method (Supplementary file 1). Sampling occurred between May 2021 and February 2022 in both health centers and gynecology offices continuously until the sample size was reached. Informed consent was obtained by the research team from participants after providing information about the purpose of the research and the study method and assuring participants’ confidentiality at all times.

### Inclusion and exclusion criteria

Participants who met the inclusion criteria were pregnant (*≤* 14 weeks gestation), aged between 18 and 45 years, and Iranian residents of Shiraz city with the ability to complete the questionnaire. Participants were excluded if they did not complete their questionnaire.

### Study sample

To determine the minimum sample size required to estimate the receipt of preconception care at a 95% confidence level and with the accuracy of estimation d = 0.03 and considering that the receipt of PCC in Shiraz city is 20% [35], after quantification in the following formula, the minimum required sample size was estimated to be *n* = 685.

$$n\, = \,{{Z_{1\, - \,{\raise0.7ex\hbox{$\alpha $} \!\mathord{\left/{\vphantom {\alpha 2}}\right.\kern-\nulldelimiterspace}\!\lower0.7ex\hbox{$2$}}\,}^2pq} \over {{d^2}}}\, = \,{{{{1.96}^2}\, \times \,0.2\, \times \,0.8} \over {{{0.03}^2}}}\, = \,685\, \approx \,693$$

#### Outcome measures and measurements

Data collection tools included three questionnaires measuring (1) individual and fertility characteristics, (2) PCC information, and (3) health literacy of Iranian adults (HELIA) aged 18 to 65 years.

1. **Questionnaire of individual and fertility characteristics** included: age, education level, employment status, insurance status, socio-economic level, marital status, number of pregnancies, and source of receiving health information, current status of pregnancy, pregnancy gestation, and method of contraception.
2. **PCC information questionnaire** includes: PCC, provider of PCC, folic acid supplement consumption prior to pregnancy, exercise, blood testing, dental care access, genetic counseling, Papanicolaou (Pap) smear testing and preconceptual vaccination uptake [1, 10, 18, 19].
3. **Health literacy of Iranian adults (HELIA) aged 18 to 65 years**: HELIA was designed and psychometrically evaluated by Montazeri et al. (2013). The questionnaire has 33 items in 5 domains, including access to information (6 items), reading skill (4 items), understanding (7 items), appraisal (4 items) and the decision-making/behavioral intention dimension with 12 items. Scores obtained from this questionnaire for each person range between 33 and 165. The literacy level score for each person ranges between 0 and 100, whereby a higher score is indicative of a higher level of health literacy. A score between 0 and 50 is interpreted as ‘inadequate’, a score between 50.1 and 66 is ‘problematic’, 66.1–84 is ‘sufficient’ and 84.1–100 is ‘excellent’. HELIA is a reliable and valid instrument for measuring health literacy in Iran, and it’s internal consistency is satisfactory, with Cronbach’s alpha coefficients ranging from 0.72 to 0.89 [36].

### Data analysis

**Fig. 1:**
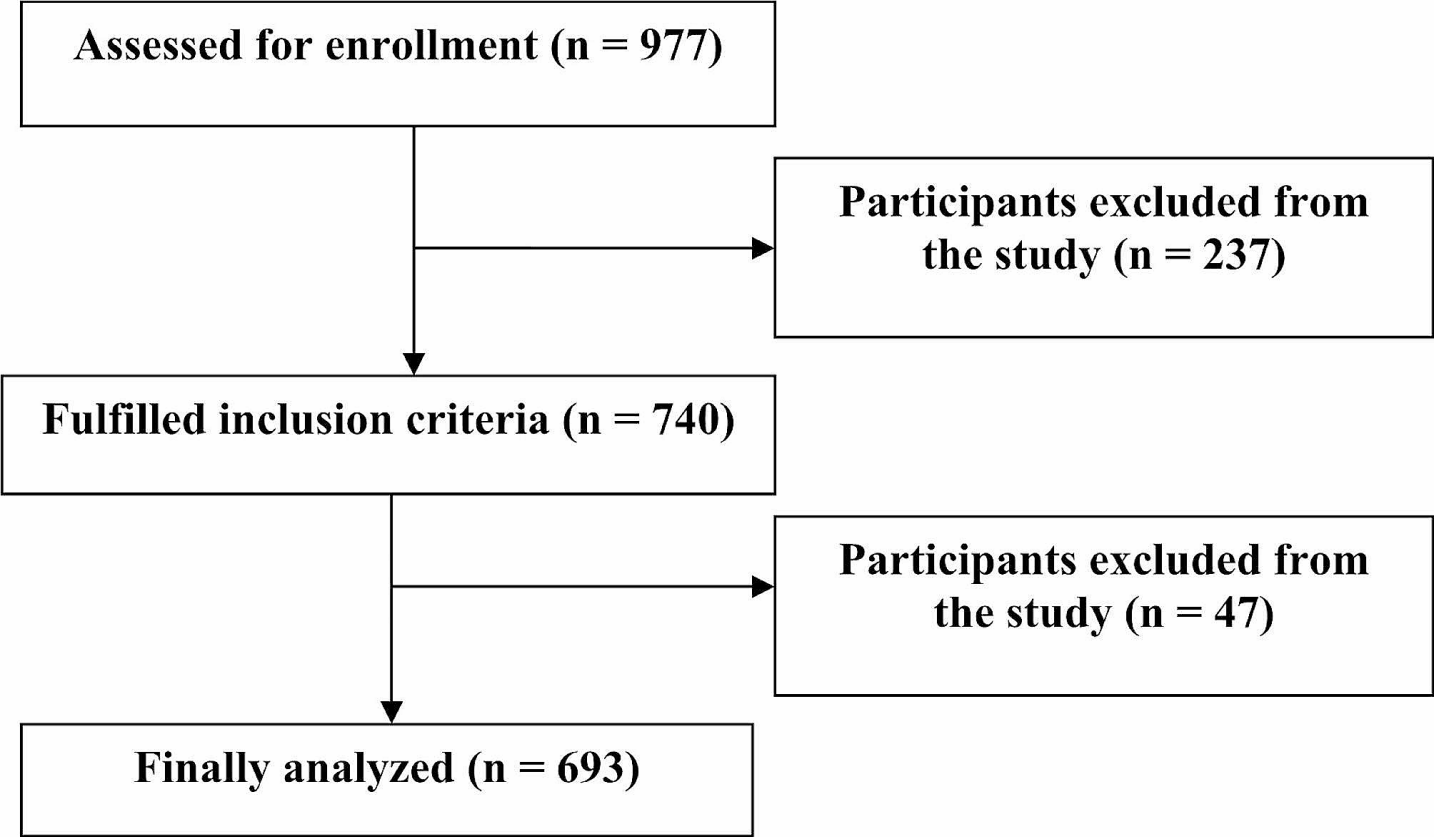
Flow chart of participant selection

Data analysis was done using SPSS version 16 software via both descriptive and inferential statistics. For descriptive statistics, frequency distributions were used for the qualitative variables and numerical indicators of minimum, maximum, average along with standard deviation for the quantitative variables. For inferential statistics, an independent sample t-test and analysis of variance were used to analyze the data. To compare the health literacy score among the components consisting of two-modes (e.g., folic acid consumption, and exercise), Student’s t-test was used, and in variables with more than two modes (e.g., place where PCC was received, blood testing, dental care, genetic counseling, Pap smear testing, and vaccination), one-way analysis of variance was used (See Fig. 1).

## Results

### Participants

As demonstrated in Table 1, an examination of the frequency of variables related to individual and fertility characteristics demonstrated that most participants were aged between 30 and 34 years old (32.2%) (Total age range: 17–44 years old), and that the majority of participants had a university level of education (59.3%), were unemployed (80.7%) with a relatively favorable self-reported economic status (58%). Overall, 78.2% of participants were of Persian ethnicity, and 56.7% had a current pregnancy between 7 and 12 weeks in gestation.

**Table 1:** Frequency distribution of individual and fertility characteristics of participants

Individuals and fertility characteristics		*n*	%
Age (years)	< 20	24	3.5
	20–24	80	11.5
	25–29	203	29.3
	30–35	262	37.8
	36–40	116	16.7
	41–45	8	1.2
Level of education	Primary	23	3.3
	Secondary	40	5.8
	Diploma	219	31.6
	University education	411	59.3
Occupational status	Unemployed	559	80.7
	Employed	134	19.3
Economic status	Unfavorable	138	19.9
	Relatively favorable	402	58
	Favorable	153	22.1
Insurance status	Yes	559	80.7
	No	134	19.3
Family relationship with spouse	Yes	284	41
	No	409	59
History of previous pregnancy	1	257	37.1
	2	247	35.6
	3	117	16.9
	> 4	72	10.4
Pregnancy planning status	Planned	570	82.3
	Unplanned	123	17.7
Previous use of family planning	Yes	397	57.3
	No	269	42.7
Method of Contraception	Hormonal methods	41	10.3
	Withdrawal	228	57.4
	Condom	113	28.5
	IUD	15	3.8
Gestational age (Weeks)	≤ 6	64	9.2
	7–12	393	56.7
	12–14	236	34.1
Source of health information	Health care providers	463	66.8
	Friends and relatives	117	16.9
	National media (including TV/radio/newspaper)	60	8.7
	Reading books, pamphlets, educational and promotional brochures & Newspapers, periodicals and magazines	137	19.8
	Social media (including WhatsApp/Telegram/Instagram, etc.)	293	42.3
	I don’t know where to get the information	37	5.3

### Health literacy and domains

As shown in Tables 2 and 40.3% of participants scored ‘sufficient’ in the access to information dimension, 38.4% scored ‘sufficient’ in the appraisal dimension, and 44.7% scored ‘sufficient’ in the decision-making and behavioral intention dimension more than any other level. However, in the two dimensions related to reading and understanding, participants had a high level of health literacy (37.7% and 58.9%, respectively), more frequently than any other level. Overall, 49.4% of participants demonstrated a ‘sufficient’ level of health literacy in this context.

**Table 2:** Frequency distribution of health literacy and domains

Health literacy and its dimensions	Inadequate		Problematic		Sufficient		Excellent	
	*n*	%	*n*	%	*n*	%	*n*	%
Access to information	137	19.8	112	16.2	**279**	**40.3**	165	23.8
Reading	81	11.7	93	13.4	258	37.2	**261**	**37.7**
Understanding	27	3.9	57	8.2	201	29	**408**	**58.9**
Appraisal	106	15.3	112	16.2	**266**	**38.4**	209	30.2
Decision making/ behavioral intention	39	5.6	113	16.3	**310**	**44.7**	231	33.3
Health literacy	21	3	111	16	**342**	**49.4**	219	31.6

As presented in Table 3, health literacy is most highly scored within the ‘understanding’ dimension with a mean of 83.84 and a standard deviation of 15.22. The lowest scores relate to the ‘access to information’ dimension with a mean of 70.14 and a standard deviation of 20.5. Health literacy obtained a mean of 76.81 with a standard deviation of 13.22.

**Table 3:** Numerical indicators of health literacy

Health literacy and its dimensions	Minimum	Maximum	Mean	SD	Base 0 to 100			
					Minimal	Maximum	Mean	SD
Access to information (6–30)	6	30	22.83	4.92	0	100	70.14	20.5
Reading (4–20)	4	20	16.35	2.96	0	100	77.24	18.59
Understanding (7–35)	11	35	30.75	4.26	14.29	100	84.83	15.22
Appraisal (4–20)	4	20	15.78	3.14	0	100	73.66	19.62
Decision making/ behavioral intention (12–60)	24	60	48.64	7.3	25	100	76.33	15.21
Health literacy (33–165)	61	165	134.38	17.45	21.21	100	76.81	13.22

### Health literacy correlates

As presented in Table 4, health literacy had a statistically significant relationship with PCC, folic acid consumption, exercise and dental care, (*p* < 0.001), along with blood testing and Pap smear testing (*p* < 0.05).

**Table 4:** Mean and standard deviation of health literacy according to PCC

PCC information		Percent (%)	Frequency (n)	Health literacy		P - value
				Mean	SD	
PCC received	Yes	463	66.8	78.45	12.26	**< 0.001** ^*^
	No	230	33.2	73.48	14.44	
Place where PCC was received	General practitioner office	75	16.2	78	13.37	0.75^**^
	Health care workers in health centers	36	7.8	76.52	11.73	
	Midwifery office	55	11.9	78.59	11.84	
	Gynecologist’s office	297	64.1	78.78	12.14	
Folic acid consumption	Yes	373	53.8	78.67	11.89	**< 0.001** ^*^
	No	320	46.2	74.63	14.34	
Exercise	Yes	316	45.6	78.41	12.09	**< 0.001** ^*^
	No	377	54.4	74.88	14.24	
Blood testing	Yes	498	71.86	77.94	12.34	**0.001** ^**^
	No	160	23.09	73.38	15.11	
	Not recommended	35	5.05	76.27	13.81	
Dental care	Yes	308	44.44	79.2	12.19	**< 0.001** ^**^
	No	348	50.21	74.94	13.59	
	Not recommended	37	5.35	74.34	14.96	
Genetic counseling	Yes	83	12	77.51	12.83	0.688^**^
	No	530	76.5	76.56	13.2	
	Not recommended	80	11.5	77.66	13.84	
Pap smear testing	Yes	370	53.4	78.01	12.92	**0.035** ^**^
	No	295	42.6	75.46	13.49	
	Not recommended	28	4	75	13.21	
Vaccination (DT, HBS, Rubella)	Yes	75	10.83	79.55	14.75	0.156 ^**^
	No	518	74.74	76.4	12.65	
	Not recommended	100	14.43	76.83	14.76	

* Independent t-test, ** One way ANOVA

## Discussion

The present study examined the relationship between health literacy and PCC. Results demonstrated that the mean health literacy of participants (76.81%) and health literacy had the highest mean score in the dimension of ‘understanding’, and the lowest mean score in the dimension related to ‘access to information’ when compared to others. Health literacy was limited in 19% of participants, (‘inadequate’ 3% and ‘problematic’ 16%). Contrariwise, health literacy was good in 81% of participants (‘sufficient’ 49.9% and ‘excellent’ 31.6%). For comparison, a mean total health literacy score of 68.32% (highest score related to ‘understanding’ and lowest score related to the dimension of ‘appraisal’) has previously been reported for Iranian adults aged 18 to 65 years living in the cities of Iran [29]. In contrast, the pregnant participants in the present study were between 18 and 45 years old, their mean score of health literacy and the level of health literacy were higher. Yet unemployed people, and those over 55 years old and people with 1 to 5 years of education have reportedly lower health literacy elsewhere [29], and so this may explain some of the difference noted. In another study including participants who had recently given birth, health literacy was reported to be relatively favorable [37]. This suggests that there may be nuanced understandings and opportunities in relation to the health literacy of Iranian childbearing populations in particular to explore.

A seperate study conducted in Iran exploring the relationship between health literacy and physical self-efficacy and including participants in the postpartum period identified that 27.5% had sufficient health literacy [38]. This is in distinct contrast to the results of the present study. Yet the rapid estimate of adult literacy in medicine tool was used in contrast to the present study, and participant numbers were much lower (*n* = 120). In another Iranian study conducted with a higher number of pregnant participants (*n* = 775) (Busher, Ahvaz, Bandar Abbas and Zahedan), results showed that despite the average age of participants being 31.89 years and similar to the present study, 15.5% had insufficient health literacy, 41.7% had borderline health literacy and 42.8% had sufficient health literacy [39]. Whilst the picture of health literacy and childbearing in Iran remains complex, differing health literacy levels are also noted elsewhere such as in Turkey, where only 33.9% of pregnant participants had a sufficient level of health literacy [40]. Considering the above, studies with larger cohorts which cover a variety of geographical areas may improve understanding in this area overall.

In relation to educational level, only 37% of participants living on the Myanmar-Thailand border were found to have sufficient health literacy despite the fact that more than half (63.1%) were able to read [41]. In the current study, approximately 60% of participants had university education, while in Gilder’s study (2019), approximately half of the studied population had no education. This could be the reason for the lower level of health literacy in such immigrant populations. In the comparisons between the present study and the studies conducted in different cities of Iran and other parts of the world, differences were notable. Seemingly, ethnicity and local culture can affect a person’s health and health literacy. Other researchers have proposed a variety of explanations for the differences seen between provinces and countries [32]. There are also differences to be noted in the measurement tools used, the uniqueness of each research population, the method of implementation, the sampling type (probability or non-probability), the method of data collection (interview or self-report), which may also affect the results of the studies and lead to differences in the health literacy scores of individuals. A standardized approach to this area of research may avoid unnecessary inconsistencies, and coupled with more qualitative approaches, enable a richer understanding of the context in which studies are being conducted.

Regarding the frequency of receiving PCC components and the relationship between health literacy and receiving PCC components, the results of this study showed that the frequency of preconceptual counseling, folic acid supplement consumption, exercise, blood testing, dental visits, genetic counseling, Pap smear testing and rubella, diphtheria, and hepatitis vaccinations prior to pregnancy was 66.8%, 53.8%, 45.6%, 71.86%, 44.44%, 12%, 53.4%, 10.83%, respectively. Many (> 64%) received PCC at specialist gynecology offices. In the current study, among the components of PCC, the highest frequency related to pre-pregnancy blood testing, and the lowest was related to receiving vaccines. Overall, 66.8% of participants received their health information from health care providers. We identified a statistically significant relationship between health literacy and PCC, taking folic acid supplements, performing blood tests, going to the dentist, and performing a Pap smear test. Ultimately, the mean score of health literacy in participants who received these components was higher than the group that did not. This suggests that those who had higher health literacy received more preventive measures and health care, a finding consistent within the literature elsewhere [33]. Indeed, people with low health literacy are less likely to receive PCC and participate in taking folic acid supplements and smoking cessation [31]. They are also less likely to receive PCC, including counseling, and prenatal vaccines [32]. Yet those who have a higher awareness of PCC have an increased chance of engaging in such preventative measures [42–45]. Thus, future healthcare strategies could usefully seek to increase uptake and knowledge of PCC alongside health literacy to increase uptake of preventative healthcare measures overall prior to pregnancy.

In the present study, the health literacy score of those who exercised before pregnancy was higher than those who did not. Among the factors of awareness, attitude, abstract norms and enabling factors, awareness is known to have the greatest impact on physical activity [46]. Thus, the first step in improving the level of physical activity is to increase awareness about the importance and correct form in physical activity. Increasing awareness in this way is likely to lead to behavior change [46]. Moreover, health literacy can promote healthier lifestyles [47], as those who have higher health literacy exercise more [47]. Thus, the challenge for future research will be to ascertain how people of reproductive age may best increase their health literacy and physical activity for healthier lives and improved outcomes in health.

Again, our results demonstrate that health literacy was higher in people receiving dental services. Consistent with this, oral health literacy has been found related to periodontal status [48], health literacy and outcomes have also found to be related to oral health and oral hygiene behaviors [49, 50]. Yet in another study conducted in Japan, health literacy was found to have no statistically significant relationship with dental visits [34]. Yet this may be because in this study, the health checkup tool was used to measure health literacy, which measures people’s health literacy in two dimensions, communicative and critical health literacy [51]. Regarding the relationship between health literacy and Pap smear testing, health literacy has been shown to be related to cancer screening in non-working people [34]. This finding is also consisted with those presented here, and suggests that where people have higher literacy levels, engagement with screening and preventative services may increase. Contrariwise, where health literacy is low, services are placed under increased pressure as people are more likely to be readmitted to healthcare units and attend emergency departments more frequently [52–54].

### Strengths and limitations and suggestions for future research

A key strength of the present study is that it has employed multi-stage sampling techniques and has achieved a high sample size. Nevertheless, there have been some non-completions of the questionnaire by participants living in more rural areas. The results presented are not generalisable. Moreover, as over half of those participating had university education, our sample may not be representative of the population of reproductive age in Shiraz. Also, the components of PCC were measured as ‘yes’ or ‘no’ rather than in more nuanced ways, and neither the number of days of folic acid consumption nor the amount of exercise taken per week was investigated. Due to the COVID-19 pandemic at the time of sampling, the majority of participants we collected data from were referred to private gynecology offices. Consequently, it was not possible to include larger numbers of participants from health centers for comparison. Future research could usefully include participants from rural areas and health centers to examine such relationships further, including those between health literacy and regular consumption of folic acid and exercise prior to pregnancy.

## Conclusion

Overall, our results demonstrate that despite health literacy being optimal, uptakes of some components of PCC are low. As such, it will be important to further raise awareness of the importance of PCC for people prior to pregnancy as a priority in health promotion and education.

### Electronic supplementary material

Below is the link to the electronic supplementary material.

Supplementary Material 1

## Data Availability

The datasets generated and analyzed during the current study are not publicly available due to the confidentiality of information, but they can be available through the corresponding author on reasonable request.
