# Supplementary material for: Investigating the relationship between health literacy and preconceptual care components during the first 14 weeks of pregnancy: a cross-sectional study
Source: BMC Prim Care. 2024 Jun 11;25:209. doi: 10.1186/s12875-024-02467-5 (PMC11165771; doi:10.1186/s12875-024-02467-5)
Supplement: Supplementary file 1 — Supplementary Material 1 [file 12875_2024_2467_MOESM1_ESM.docx]

**Supplementary file 1: Frequency distribution of the sampling location in participants referring to health centers and gynecology offices**

| **Sampling location** | **Frequency**  **(n)** | **Percent**  **(%)** |
| --- | --- | --- |
| Health centers | 144 | 20.8 |
| Gynecology offices | 549 | 79.2 |
| Total | 693 | 100 |
